# Supplementary material for: Gut Microbiota Ecological and Functional Modulation in Post-Stroke Recovery Patients: An Italian Study
Source: Microorganisms. 2023 Dec 25;12(1):37. doi: 10.3390/microorganisms12010037 (PMC10819831; doi:10.3390/microorganisms12010037)
Supplement: Supplementary file 1 [file microorganisms-12-00037-s001.zip › Supplementary Files/Supplementary Figure/Figure S1.pdf]

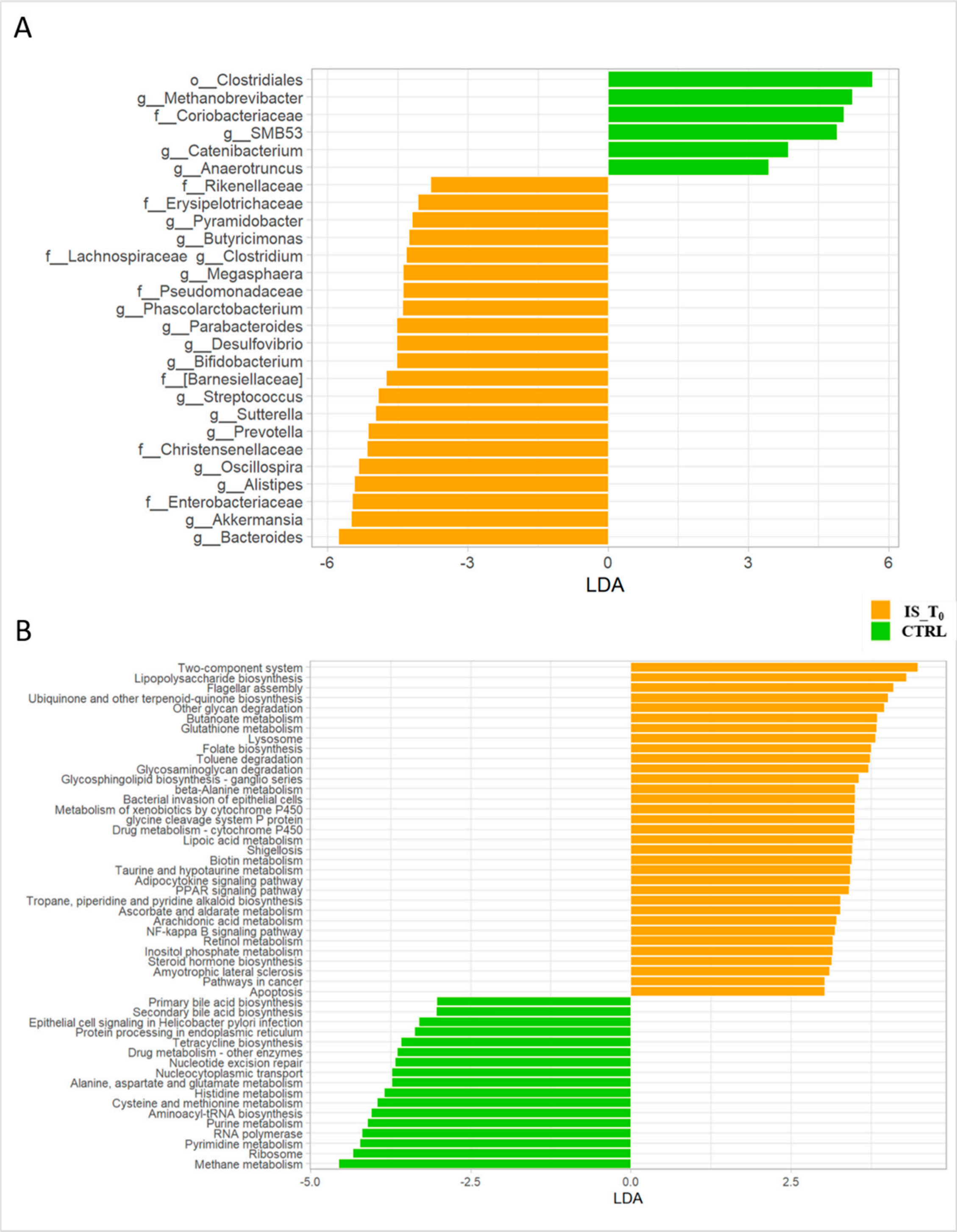

Figure S1: (A) LEfSe analysis for the comparison between the IS patients at T_0 and CTRLs groups' GM. Histogram represents the LDA scores until L6 taxonomy filtered by statistical significance between the two groups. In red and in green are represented microbial biomarkers for the CTRLs subjects and IS patients, respectively; (B) Kyoto Encyclopedia of Genes and Genomes (KEGG) biomarkers inferred from the whole set of 46 ASVs of the IS patients at T_0 and the CTRLs. Linear discriminant analysis (LDA) effect size (LEfSe) was performed on the PICRUSt2 predicted biochemical pathways matrix. The reported pathways were filtered for statistically significance and LDA ± 3.0.

IS_ T_0: IS patients at T_0; CTRL: controls subjects; LDA: Linear Discriminant Analysis score.
