# Supplementary material for: Gut Microbiota Ecological and Functional Modulation in Post-Stroke Recovery Patients: An Italian Study
Source: Microorganisms. 2023 Dec 25;12(1):37. doi: 10.3390/microorganisms12010037 (PMC10819831; doi:10.3390/microorganisms12010037)
Supplement: Supplementary file 1 [file microorganisms-12-00037-s001.zip › Supplementary Files/Supplementary Figure/Figure S2.pdf]

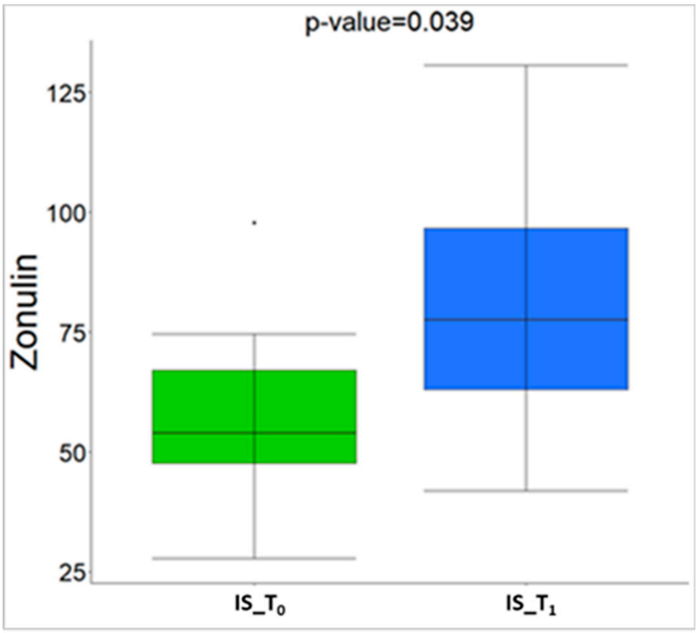

Figure S2: Distribution of Zonulin in IS patients T_0 and IS patients T_1.
T-test p-value ≤ 0.05.
IS_T_0: IS patients at T_0; IS_T_1: IS patients at T_1; CTRL: controls subjects.
