# Supplementary material for: Gut Microbiota Ecological and Functional Modulation in Post-Stroke Recovery Patients: An Italian Study
Source: Microorganisms. 2023 Dec 25;12(1):37. doi: 10.3390/microorganisms12010037 (PMC10819831; doi:10.3390/microorganisms12010037)
Supplement: Supplementary file 1 [file microorganisms-12-00037-s001.zip › Supplementary Files/Supplementary Figure/Figure S3.pdf]

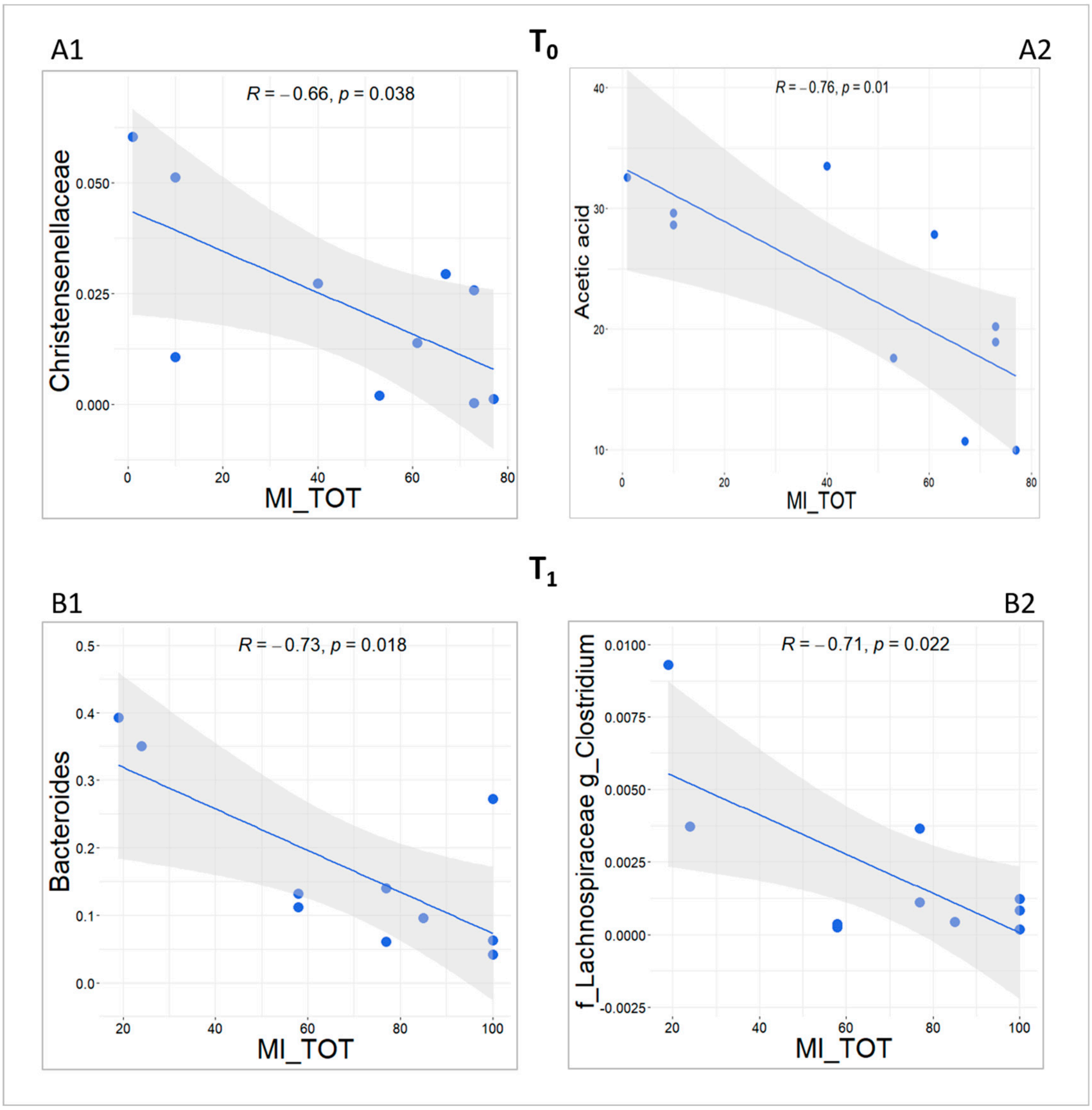

Figure S3: Significant Pearson correlations between ASVs and SCFAs at T_0 (A) and T_1 (B) with motricity index (MI_TOT). (A1) Christensenellaceae; (A2) Acetic acid; (B1) *Bacteroides*; (B2) *Clostridium* (Lachnospiraceae).
Only statistically significant (*p*-value ≤ 0.05) correlations between ASVs, SCFAs and MI_TOT were showed.
